# Autonomic versus perceptual accounts for tactile hypersensitivity in autism spectrum disorder

**DOI:** 10.1038/s41598-017-08730-3

**Published:** 2017-08-15

**Authors:** Hiroshi Fukuyama, Shin-ichiro Kumagaya, Kosuke Asada, Satsuki Ayaya, Masaharu Kato

**Affiliations:** 1 Center for Baby Science, Doshisha University, Kizugawa, Japan; 2 Graduate School of Arts and Sciences, The University of Tokyo, Tokyo, Japan; 3 Research Center for Advanced Science and Technology, The University of Tokyo, Tokyo, Japan

## Abstract

Tactile atypicality in individuals with autism spectrum disorder (ASD) has harmful effects on their everyday lives including social interactions. However, whether tactile atypicality in ASD reflects perceptual and/or autonomic processes is unknown. Here, we show that adults with ASD have hypersensitivity to tactile stimuli in the autonomic but not perceptual domain. In particular, adults with ASD showed a greater skin conductance response (SCR) to tactile stimuli compared to typically developing (TD) adults, despite an absence of differences in subjective responses. Furthermore, the level of the SCR was correlated with sensory sensitivity in daily living. By contrast, in perceptual discriminative tasks that psychophysically measured thresholds to tactile stimuli, no differences were found between the ASD and TD groups. These results favor the hypothesis that atypical autonomic processing underlies tactile hypersensitivity in ASD.

## Introduction

Autism spectrum disorder (ASD) is a neurodevelopmental disorder characterized by problems with interpersonal interaction and communication and restricted or repetitive behaviors. Studies have demonstrated that many children and adults with ASD have also sensory difficulties in various modalities^1–12^. In addition, individuals with ASD have reported sensory difficulties in their autobiographies (see also a meta-analysis of autobiographies by individuals with ASD^13^). In line with these reports, sensory atypicality has recently been added to the diagnostic criteria for ASD in the Diagnostic and Statistical Manual, fifth edition (DSM-5)^14^. However, the underlying mechanisms of sensory atypicality in individuals with ASD are poorly understood. In particular, literature on tactile processing in ASD is sparse compared to that for other modalities such as visual and auditory processing^15^. Because tactile atypicality affects everyday life by leading to broad difficulties in typical life activities, such as impairments in selecting clothes, participating in crowded assemblies, and building intimate relationships (e.g., parent-child bonding and romantic partnership), better elucidating the mechanisms of tactile atypicality in ASD will inform efforts to improve the quality of everyday life in individuals with ASD.

Traditionally, research has accumulated knowledge about sensory atypicality mostly by relying on subjective reports by individuals with ASD or their parents and teachers; in this decade, experimental studies have examined perceptual sensitivity to tactile stimuli in ASD more objectively with a psychophysical approach (for detail, see a recent review^16^). Speaking tentatively, studies have consistently demonstrated that individuals with ASD are not different from typically developing (TD) controls in detection of light touch^17,18^, but results on potential differences in thresholds to various vibrotactile stimulations have been inconsistent^17,19–22^. Moreover, some studies have demonstrated dysfunction of adaptation to continuous tactile stimulation in ASD^21,23–25^ with one exception^17^. These inconsistent results on perceptual processing in ASD are partly due to variability in body sites stimulated, measurements, types of stimuli, and participants’ ages^2,16^. Although some studies investigated detection thresholds in multiple body sites (e.g., finger/palm and forearm^17,19^), investigation of the neck would provide new insight on tactile process in ASD because anecdotal reports suggest that turtlenecks or clothing tags are uncomfortable for individuals with ASD.

Furthermore, sensitivity of somatotopic maps which normally vary with body sites^26^ has never been investigated in ASD. However, a magnetoencephalography (MEG) study suggests atypicality in somatosensory cortex of adults with ASD^27^. Although previous studies have consistently reported that there was no group difference in the detection of light touch, studies have never investigated sensitivity to slight differences between tactile stimuli. Moreover, the interpretation of the results of this work is complicated because the manifestation of hypersensitivity might be bidirectional. While hypersensitivity to tactile stimuli would lead to lower thresholds in a detection task^17,19^, it could hinder detection and discrimination, possibly through impairment of inhibitory processes^21,22^. The mixed and bidirectional results of these experimental studies raise the possibility that aspects other than perceptual processing may also lead to atypical tactile experience in ASD.

Given the tactile difficulties experienced in daily living, affective responses to tactile stimuli may be atypical in ASD^20^. If affective responses to tactile stimuli are excessive, tactile stimuli may make people have difficulties in relaxing, concentrating on experimental tasks, or sharing their experiences with other people. Affective responses to tactile stimuli have been investigated using subjective reports^17,19^. Blakemore, *et al*.^19^ showed that adults with ASD rated tactile stimuli to be more tickly and intense than did adult control participants. Cascio, *et al*.^17^ demonstrated that adults with ASD felt increased thermal pain relative to control. These measurements have characterized subjective experiences in ASD, but underlying mechanisms leading to atypicality in affective responses in ASD are still unknown.

Previous studies have suggested that individuals with ASD have an atypical autonomic nervous system^28–30^. The autonomic nervous system consists of the sympathetic (fight or flight) and parasympathetic (rest and digest) nervous systems and is thought to function in emotion regulation by regulating homeostasis. In a social context, children with ASD showed larger skin conductance responses (SCR), a measurable readout of sympathetic nervous system activity, to face stimuli than did TD controls^31^. One previous study demonstrated that children with ASD showed lower baseline SCR and decreased SCR in response to tactile stimuli than those of TD^32^. However, because this study did not investigate the children’s subjective feelings to tactile stimuli, it failed to find a relationship between autonomic responses and sensory difficulties in daily lives. Although these studies have investigated participants’ autonomic response during mentally demanding cognitive tasks or social stimuli presentation, it is possible that individuals with ASD show excessive autonomic responses to sensory stimuli apart from cognitive tasks and regardless of social/non-social context.

To address these questions on the nature of affective response atypicality, we investigated autonomic responses to tactile stimuli as well as subjective feelings. We also aimed to investigate tactile discrimination at various body sites to extend previous findings on perceptual processing in ASD. To achieve these goals, we conducted four tasks using a within-participant design. To investigate affective sensitivity, we measured autonomic response by recording SCR to electrical tactile stimuli and determined participants’ subjective responses. The other three tasks measured sensory thresholds to a variety of discriminative tactile perceptions using psychophysical methods. First, we measured participants’ sensitivity related to somatotopic mapping through a two-point discrimination task. Second, we measured their ability to detect a small pressure using a set of thin nylon “von Frey” monofilaments. Third, to examine the possibility that individuals with ASD detect slight differences between tactile stimuli, we measured participants’ ability to distinguish different pressures applied via these monofilaments. Through these investigations, we were able to better clarify what causes tactile atypicality in ASD. In particular, we clarified whether individuals with ASD exhibit hypersensitivity to tactile stimuli in autonomic and perceptual processes.

## Results

### Electrical stimulation task

#### Autonomic response

We first analyzed the fidelity and consistency of the electrical stimulation task across participants and groups. Two participants with ASD could not undergo the electrical stimulation task owing to a scheduling conflict. One participant with ASD refused the stimulation owing a low tolerance threshold, as described in the methods. One TD participant was excluded from the analysis because of an experimental error. We defined a peak (maximum value) of SCR within 10 sec after the stimulus offset as an event-related SCR. The mean latency of the peak was 4.593 sec with 1.883 SD. And we included participants who showed this peak in response to the weak and strong intensity stimuli in at least one trial. Four participants with ASD and three TD participants were excluded for failure to meet these criteria. Thus, 16 participants with ASD and 15 TD participants were included in the final analysis.

To confirm the intensity of stimuli (i.e., electrical current) presented to participants was not different between the groups, we conducted a mixed measures ANOVA using group (ASD versus TD) as a between-participant factor and stimulus intensity (weak versus strong) as a within-participant factor (ASD_weak_ M = 400 μA, SD = 133; TD_weak_ M = 376 μA, SD = 90; ASD_strong_ M = 1209 μA, SD = 537; TD_strong_ M = 1352 μA, SD = 288). As expected, there was a significant main effect of stimulus intensity (*F*(1, 29) = 173.775, *p* < 0.001, partial *η* ^2^ = 0.857), but no main effect of group (*F*(1, 29) = 0.419, *p* = 0.523, partial *η* ^2^ = 0.014), nor was there an interaction between group and stimulus intensity (*F*(1, 29) = 1.533, *p* = 0.226, partial *η* ^2^ = 0.05). Thus the magnitude of electrical current presented to participants was not significantly different between the two groups.

To determine whether there was a group difference in the baseline skin conductance levels irrespective of electrical stimulation, a mixed measures ANOVA was conducted on SCR values immediately before the stimulus onset using group (ASD versus TD) as a between-participant factor and stimulus intensity (weak versus strong) as a within-participant factor (ASD_weak_ M = 5.977 μS, SD = 2.387; TD_weak_ M = 5.008 μS, SD = 2.405; ASD_strong_ M = 5.821 μS, SD = 2.427; TD_strong_ M = 5.048 μS, SD = 2.432). There were no significant differences between the groups (*F*(1, 29) = 1.015, *p* = 0.322, partial *η* ^2^ = 0.034), nor were there significant differences in the stimulus intensities (*F*(1, 29) = 0.744, *p* = 0.395, partial *η* ^2^ = 0.025). There was no interaction between group and stimulus intensity (*F*(1, 29) = 2.169, *p* = 0.152, partial *η* ^2^ = 0.070). This analysis confirmed that the two groups did not differ in the baseline skin conductance levels.

**Figure 1:**
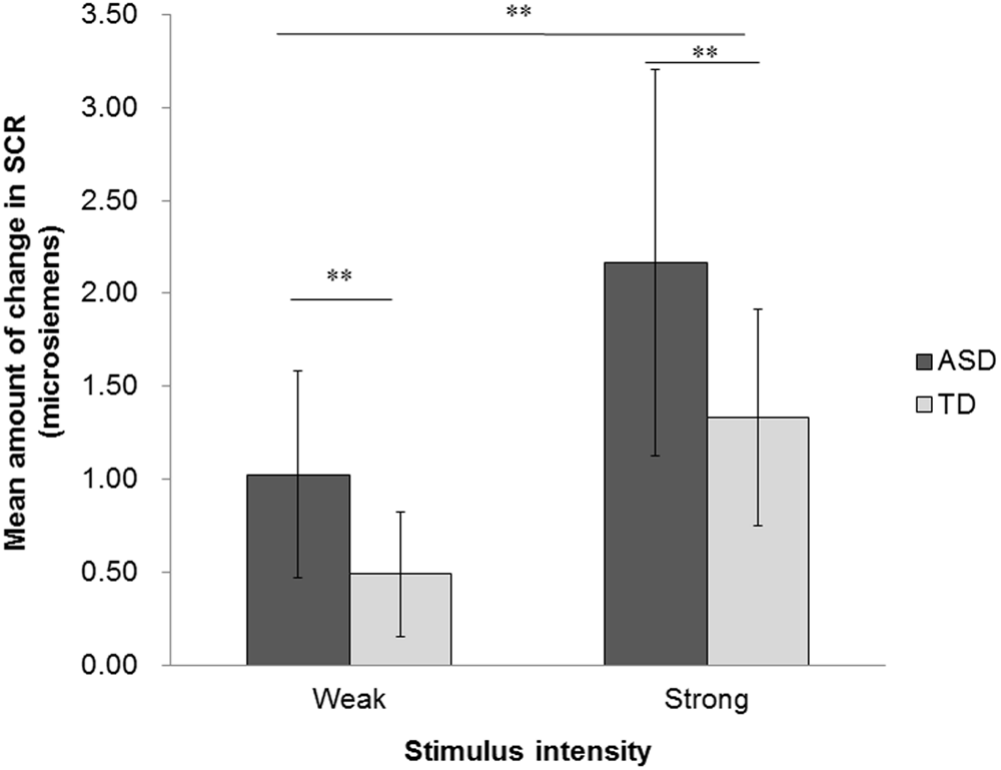
Mean amount of change in the SCR to electrical stimuli across groups as a function of stimulus intensity. Error bars show SD. **p < 0.01.

Having confirmed that there were no group differences in the intensity of stimuli and baseline SCR (i.e., SCR in absence of stimulation), we next analyzed stimulus event related SCR in each group to determine if there were differences between the ASD and TD groups in autonomic responses. The event-related SCR was computed by subtracting the value immediately before the stimulus onset from the peak value after the stimulus offset for each trial. We then calculated the mean SCR difference at each stimulus intensity and for each participant. Figure 1 shows the mean event-related SCR to the electrical stimulus for each group. A mixed measures ANOVA was conducted on the event-related SCR using group (ASD versus TD) as a between-participant factor and stimulus intensity (weak versus strong) as a within-participant factor (ASD_weak_ M = 1.025 μS, SD = 0.559; TD_weak_ M = 0.489 μS, SD = 0.336; ASD_strong_ M = 2.165 μS, SD = 1.040; TD_strong_ M = 1.332 μS, SD = 0.581). The analysis revealed significant main effects of group (*F*(1, 29) = 9.032, *p* = 0.005, partial *η* ^2^ = 0.237) and of stimulus intensity (*F*(1, 29) = 112.567, *p* < 0.001, partial *η* ^2^ = 0.795). However, no significant interaction between group and stimulus intensity was found (*F*(1, 29) = 2.519, *p* = 0.123, partial *η* ^2^ = 0.08).

To evaluate relationships between the symptoms of ASD and autonomic reactivity, we next conducted correlation analyses on the subcategories of the Social Responsiveness Scale, second edition (SRS-2), and the event-related SCR at each stimulus intensity by calculating Pearson’s correlation coefficients. Controlling the false discovery rate at 0.05 using the Benjamini-Hochberg procedure^33^ for this multiple comparison produced the same level of significance as defined based on *p*-values. The critical values of this procedure were reported as *q*-values. The *q*-value was defined as *q* = *Q* * *i*/*m*, where *i* was the rank of *p*-value when the *p*-values were arranged in ascending order, *m* was the total number of tests, and *Q* was the false discovery rate, which was set to 0.05 in the current study. The largest *p*-value for which *p* < *q*, and all of the *p*-values smaller than this, were considered statistically significant. All subcategories of the SRS-2 were positively correlated with the SCRs at both weak and strong intensities with the exception of Social Motivation in the strong intensity (Social Awareness: weak *r* = 0.558, *p* = 0.001, *q* = 0.018, strong *r* = 0.392, *p* = 0.029, *q* = 0.039; Social Cognition: weak *r* = 0.517, *p* = 0.003, *q* = 0.025, strong *r* = 0.381, *p* = 0.034, *q* = 0.043; Social Communication: weak *r* = 0.670, *p* < 0.001, *q* = 0.004, strong *r* = 0.452, *p* = 0.011, *q* = 0.029; Social Motivation: weak *r* = 0.581, *p* = 0.001, *q* = 0.014, strong *r* = 0.338, *p* = 0.063 (not significant), *q* = 0.050; Restricted Interests and Repetitive Behavior: weak *r* = 0.530, *p* = 0.002, *q* = 0.021, strong *r* = 0.372, *p* = 0.040, *q* = 0.046; Social Communication and Interaction: weak *r* = 0.642, *p* < 0.001, *q* = 0.007, strong *r* = 0.430, *p* = 0.016, *q* = 0.032; Total: weak *r* = 0.629, *p* < 0.001, *q* = 0.011, strong *r* = 0.425, *p* = 0.017, *q* = 0.036).

To investigate the relationship between participants’ sensory experiences in their daily lives and autonomic reactivity observed in the laboratory, we conducted correlation analyses (Pearson’s correlation coefficient) on the subcategories of the Adolescent/Adult Sensory Profile (AASP) and the event-related SCR for each stimulus intensity. The Benjamini-Hochberg procedure^33^ for this multiple comparison indicated the same level of significance, as defined based on *p*-values. For the weak intensity, Sensitivity to Stimuli was positively correlated with SCR, *r* = 0.459, *p* = 0.009, *q* = 0.006, whereas Sensation Seeking was negatively correlated with SCR, *r* = −0.454, *p* = 0.010, *q* = 0.013. The other two subcategories of AASP were not significantly correlated with SCR (Poor Registration: *r* = 0.344, *p* = 0.058, *q* = 0.019; Sensation Avoiding: *r* = 0.332, *p* = 0.068, *q* = 0.031). For the strong intensity, however, there were no significant correlations between the subcategories and SCR, with all *p-*values exceeding 0.06, each of which was larger than the *q*-value.

#### Subjective response

**Figure 2:**
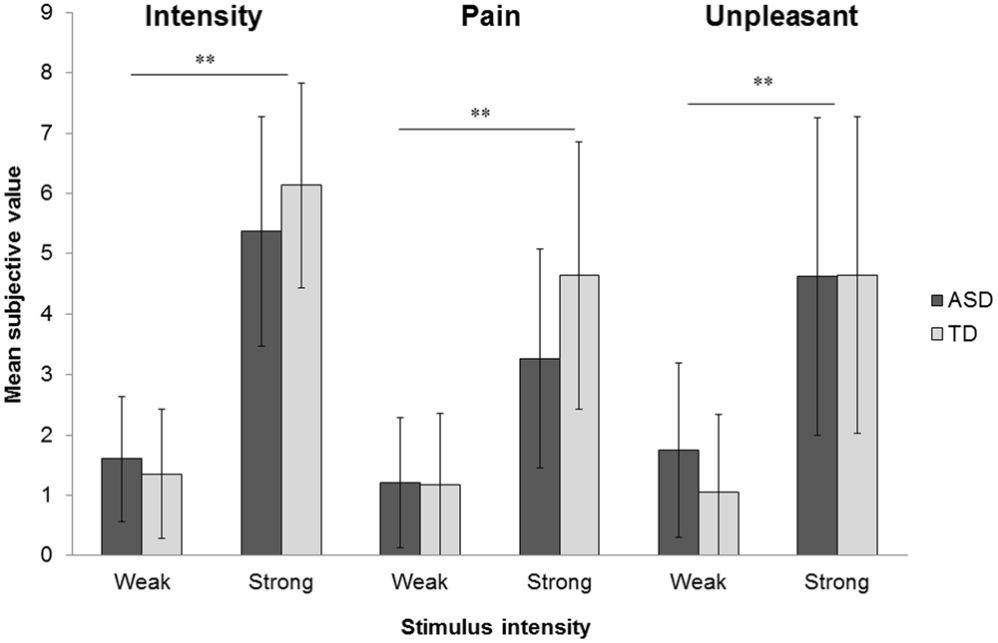
The subjective values for each perception across groups as a function of stimulus intensity. Error bars show SD. **p < 0.01.

A mixed measures ANOVA was conducted on subjective value for each category (intensity, pain, and unpleasant) using group (ASD versus TD) as a between-participant factor and stimulus intensity (weak versus strong) as a within-participant factor. The results of this analysis are displayed in Fig. 2. With respect to subjective intensity, a significant main effect of stimulus intensity was found (*F*(1, 29) = 237.112, *p* < 0.001, partial *η* ^2^ = 0.891). However, neither a main effect of group (*F*(1, 29) = 0.316, *p* = 0.578, partial *η* ^2^ = 0.011) nor an interaction between group and stimulus intensity were observed (*F*(1, 29) = 3.290, *p* = 0.080, partial *η* ^2^ = 0.102). A significant main effect of stimulus intensity was found for the pain subcategory (*F*(1, 29) = 94.451, *p* < 0.001, partial *η* ^2^ = 0.765). There was no main effect of group (*F*(1, 29) = 1.710, *p* = 0.201, partial *η* ^2^ = 0.056), but there was a significant interaction between group and stimulus intensity (*F*(1, 29) = 6.092, *p* = 0.020, partial *η* ^2^ = 0.174). To further explore this interaction, a Mann-Whitney’s *U*-test was conducted for each stimulus intensity. However, no significant group differences were found (weak: *U* = 113.5, *p* = 0.800, *z* = 0.260; strong: *U* = 164.5, *p* = 0.078, *z* = 1.765). With respect to the unpleasant category, a significant main effect of stimulus intensity was found (*F*(1, 29) = 84.367, *p* < 0.001, partial *η* ^2^ = 0.744). However, there was no main effect of group (*F*(1, 29) = 0.265, *p* = 0.610, partial *η* ^2^ = 0.009) or an interaction between group and stimulus intensity (*F*(1, 29) = 1.058, *p* = 0.312, partial *η* ^2^ = 0.035).

#### Sensation and pain threshold evaluations

We additionally sought to determine if there were differences in pain thresholds between groups. Three participants with ASD did not participate in this task due to either scheduling conflicts (n = 2) or refusal of electrical stimulation (n = 1). A two-sample *t*-test revealed there was not a group difference in the sensation threshold (ASD: M currency = 8.262 μA, SD = 1.563, TD: M = 8.375 μA, SD = 1.505, *t*(37) = 0.231, *p* = 0.818, Cohen’s *d* = 0.07). Similarly, a two-sample *t*-test indicated no difference between groups (pain threshold, ASD: M currency = 57.235 μA, SD = 41.927, TD: M = 47.814 μA, SD = 22.422, *t*(37) = 0.868, *p* = 0.391, Cohen’s *d* = 0.28).

### Two-point discrimination task

**Figure 3:**
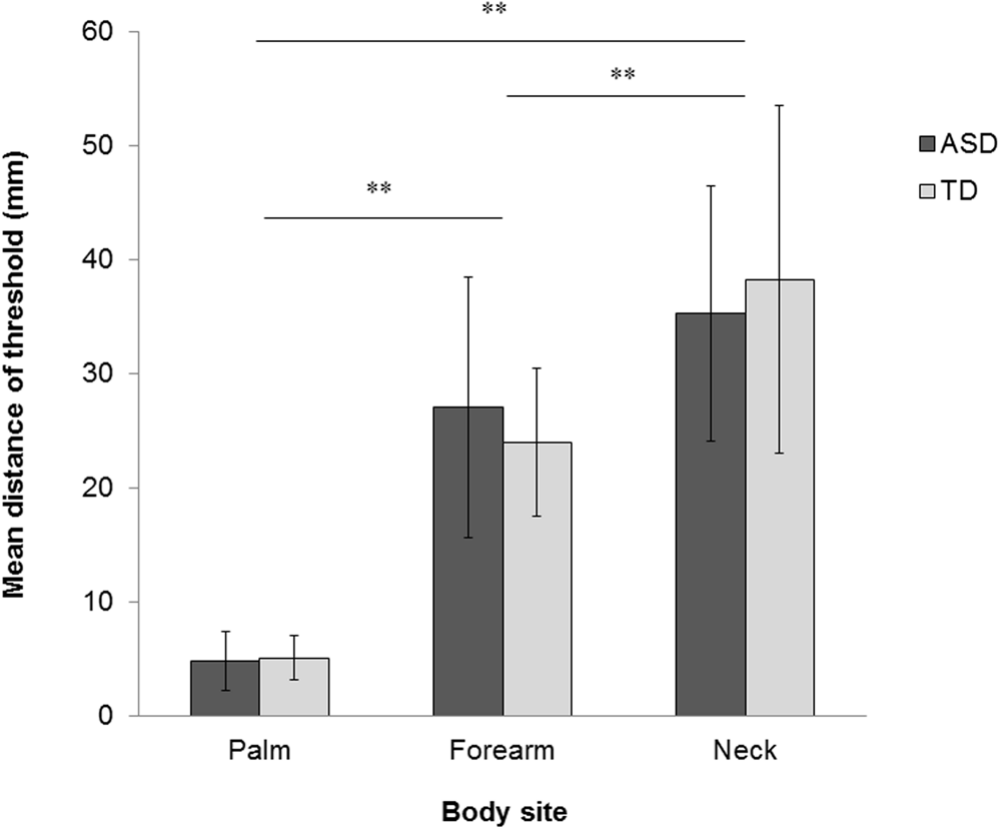
Mean of the distance of the thresholds for two point discrimination across groups as a function of body site. Error bars show SD. **p < 0.01.

To assess if there were differences in tactile acuity between ASD and TD participants, we employed a two-point discrimination task. One participant with ASD, who participated in this task, was excluded from the final analysis because of an experimental error. Figure 3 shows the mean thresholds of the touch detection task for each body site. A mixed measures ANOVA was conducted on the thresholds using site (palm, forearm, neck) as a within-participant factor and group (ASD versus TD) as a between-participant factor. The analysis revealed a significant main effect of site (*F*(2, 78) = 133.114, *p* < 0.001, partial *η* ^2^ = 0.773; ASD: palm M = 4.900 mm, SD = 2.579; forearm M = 27.145 mm, SD = 11.422; neck M = 35.318 mm, SD = 11.196; TD: palm M = 5.147 mm, SD = 1.918; forearm M = 24.074 mm, SD = 6.489; neck M = 38.316 mm, SD = 15.246). A post-hoc multiple comparison with Bonferroni correction revealed that the threshold on the palm was significantly narrower than both the forearm and the neck, and the threshold on the forearm was significantly narrower than the neck across groups (all *p*-values < 0.001). There was neither a significant main effect of group (*F*(1, 39) = 0.001, *p* = 0.976, partial *η* ^2^ < 0.001), nor a significant interaction between site and group (*F*(2, 78) = 1.182, *p* = 0.312, partial *η* ^2^ = 0.029).

### Touch detection task

**Figure 4:**
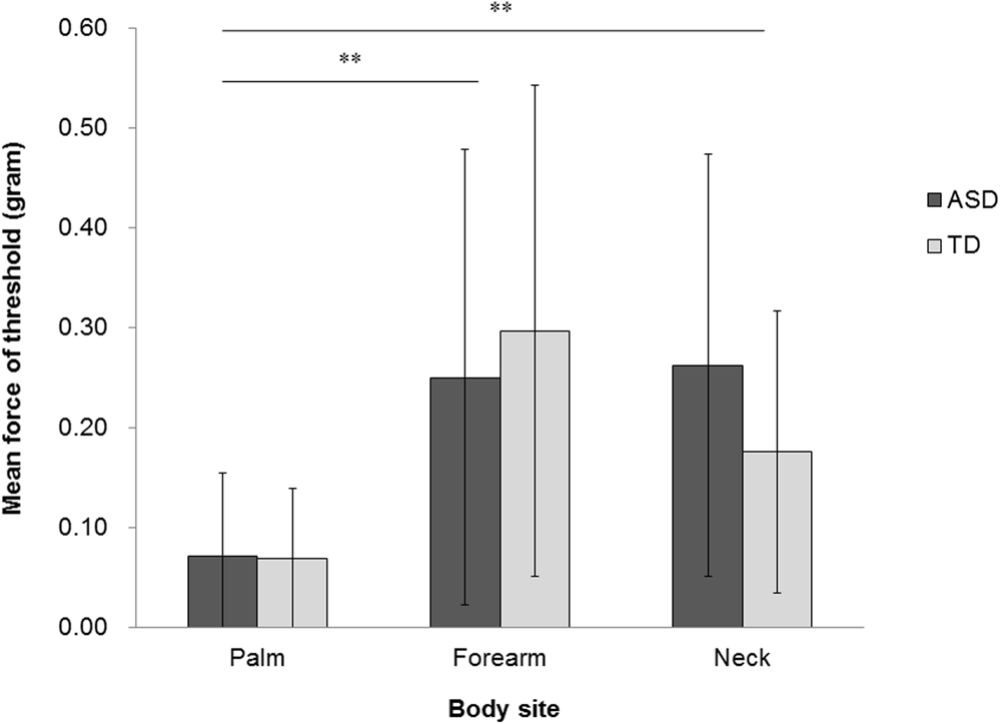
Mean of the force of the thresholds for touch detection to light touch (monofilament stimuli) across groups as a function of body sites. Error bars show SD. **p < 0.01.

We further evaluated if there were differences in touch threshold between groups. One participant with ASD participated in this task, but was excluded from the final analysis because of an experimental error. Figure 4 shows the mean thresholds of the touch detection task for each body site. A mixed measures ANOVA was conducted on the threshold values using site (palm, forearm, neck) as a within-participant factor and group (ASD versus TD) as a between-participant factor. The analysis revealed a significant main effect of site (*F*(2, 78) = 18.463, *p* < 0.001, partial *η* ^2^ = 0.321; ASD: palm M = 0.071 gram, SD = 0.083; forearm M = 0.251 gram, SD = 0.228; neck M = 0.263 gram, SD = 0.210; TD: palm M = 0.069 gram, SD = 0.070; forearm M = 0.297 gram, SD = 0.246; neck M = 0.176 gram, SD = 0.141). A post-hoc multiple comparison with Bonferroni correction revealed the threshold on the palm was significantly lower than those on the forearm and the neck (both *p-*values < 0.001). No significant difference was found between the forearm and the neck. We did not observe a significant main effect of group (*F*(1, 39) = 0.134, *p* = 0.716, partial *η* ^2^ = 0.003) or a significant interaction between site and group (*F*(2, 78) = 1.870, *p* = 0.161, partial *η* ^2^ = 0.046).

### Touch comparison task

We next assessed the sensitivity to and discernment of differences in the force of a light touch, using one of the forces employed in the touch detection task as the standard stimulus and a range of forces as comparison stimuli. In this analysis, the comparison stimulus force was the independent variable. For each comparison force, we calculated the fraction of participants who reported the stimulus to be stronger than the standard stimulus. A cumulative logistic distribution as a function of the forces (*x*) was fitted to the data using the following equation to obtain a psychometric function:

$$f(x)=\frac{1}{1+\exp (-\,x)}$$

**Figure 5:**
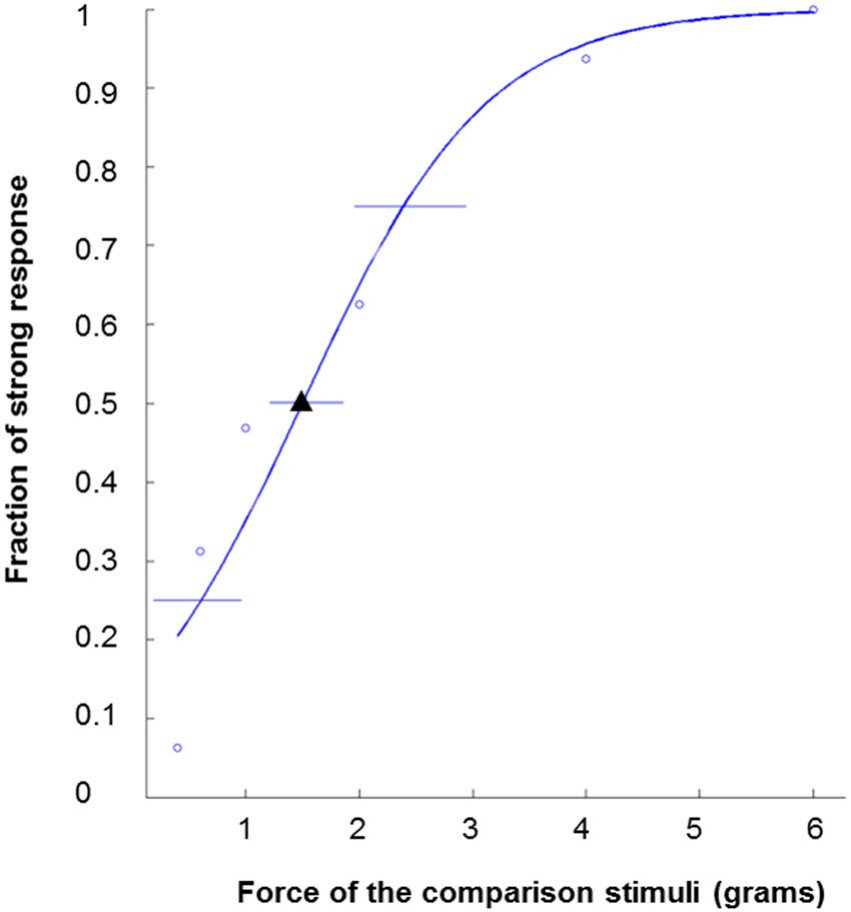
An example of the psychometric function of an ASD participant in the touch comparison task. “o”s show the fractions of the participant’s strong response as a function of the forces of the comparison stimuli when compared with the standard stimulus (1.4 g). “▴” shows the point on the curve at which the percentage of strong response was 50%. The slope (derivative) of the curve was used as the dependent variable.

To evaluate individual sensitivity to differences between the standard stimulus and the comparison stimuli, a slope (derivative) of a point representing 50% of the fraction of strong response on the curve of the psychometric function was calculated for each participant (Fig. 5). A steeper slope indicates greater sensitivity to the stimulus difference. The psychometric function was generated using MATLAB (version 7.12, MathWorks, Natick, MA) running Psignifit toolbox (psignifit_3.0beta.20120611.1, http://psignifit.sourceforge.net/).

To compare the sensitivity to force differences in light touch between the ASD and TD groups, a two-sample *t*-test was conducted on the slope (derivative). The analysis revealed there was no significant difference between the ASD and TD groups (*t*(40) = 1.281, *p* = 0.208, Cohen’s *d* = 0.40; Supplementary Fig. S1). To investigate the group difference in each stimulus, a mixed measures ANOVA was conducted on the percentage of strong responses using force of the stimulus (0.4 g, 0.6 g, 1.0 g, 2.0 g, 4.0 g, 6.0 g) as a within-participant factor and group (ASD versus TD) as a between-participant factor. There was a significant main effect of force of the stimulus (*F*(5, 200) = 670.773, *p* < 0.001, partial *η* ^2^ = 0.944). A post-hoc multiple comparison with Bonferroni correction revealed that all stimulus pairs except one were significantly different (all *p*-values < 0.001, except a pair between 4.0 g and 6.0 g, *p* = 0.107). There was neither a significant main effect of group (*F*(1, 40) = 0.347, *p* = 0.559, partial *η* ^2^ = 0.009), nor a significant interaction between stimulus and group (*F*(5, 200) = 0.881, *p* = 0.495, partial *η* ^2^ = 0.022; Supplementary Fig. S2).

## Discussion

The current study investigated affective and discriminative aspects of tactile sensitivity in ASD in order to determine if atypicality in tactile sensation in ASD is reflected in underlying autonomic and perceptual processes. To investigate affective sensitivity, we analyzed autonomic and subjective responses to electrical stimuli. The event-related SCR, which reflects sympathetic nervous activity, was significantly larger in the ASD group than in typically developing controls while there were no group differences in the pre-stimulus skin conductance level and the magnitude of the presented stimuli. Note that the presented stimuli were always suprathreshold therefore it is unlikely that the results were attributed to a possible group difference in detection threshold^21^. A relationship between increased sympathetic nervous activity and autistic symptoms was further confirmed by positive correlations between the event-related SCR and the total scores and almost all subcategory scores on the SRS-2, an index of the severity of autistic symptoms. The current study is thus the first to demonstrate excessive autonomic response to tactile stimuli in ASD.

These results however contrast with a previous study demonstrating lower SCR to tactile stimuli (feather) in ASD than TD^32^. This is probably because the type of stimulation and the situation were quite different between the studies. In contrast to our task where participants had to report their feelings to the suprathreshold stimulations, children were not required to pay attention to the stimulations during an irrelevant task in Schoen *et al*.’s study. Therefore, the possibility that the irrelevant task rather than tactile stimuli influenced autonomic states of children with ASD could not be ruled out. In the current study, we also examined various subjective responses (intensity, pain, and unpleasantness) to the tactile stimulations as well as autonomic responses. While there were significant differences in responses across stimulus intensities, no differences were observed between groups indicating that both groups could comparably discriminate the stimuli in terms of intensity. Interestingly, and in contrast to Schoen *et al*., the event-related SCR to the weak intensity stimulus was correlated with the Sensitivity to Stimuli subcategory score of the AASP. These results suggest that affective tactile hypersensitivity in ASD might not be an immediate manifestation of the subjective response to the stimulus. Instead, they suggest that the cumulative effects of excessive autonomic responses to suprathreshold tactile stimuli may lead to sensory difficulties in daily living.

Similar to subjective feelings to the suprathreshold stimulations, there were no group differences in the threshold for sensation and painfulness to electrical stimuli. In previous studies investigating detection thresholds using vibrotactile stimuli^21,22^, the threshold of dynamic tactile stimuli was not different between groups although that of constant stimuli was higher in the ASD group than in the TD group. Because the sensation task presented dynamic stimuli (i.e., the current gradually increased from zero in each trial), the results on detection threshold reported in the current study were consistent with previous studies. Taken together, our results indicate that adults with ASD subjectively feel tactile stimuli in the same way as TD adults but that their autonomic processes responding to such stimuli are excessive.

In contrast to atypicality observed in the autonomic activity, we could not find any group differences across three perceptual discriminative tasks. First, in the two-point discrimination task, both groups showed similar patterns of the thresholds at the three body sites tested, palm < forearm < neck. The mean thresholds and the standard deviations reported in the current study were comparable with those of a previous study^26^, although they could not be directly compared because the procedures were not identical. While an MEG study has suggest that individuals with ASD have atypical somatotopic mapping^27^, the current result suggests that adults with or without ASD have a similar distribution in the somatosensory area in relation to discriminative thresholds. This inconsistency may be partly due to the body sites t examined. The MEG study examined the lip and the fingertips, which are composed of hairless skin and generally cover larger somatosensory areas than the forearm and the neck. Examining broad body sites with both psychophysical and neurological techniques should resolve this inconsistency.

Second, the touch detection task also did not show any group differences, and the palm was more sensitive than the other two body sites regardless of group. This result is consistent with previous findings examining the palm^17^ and the forearm^17,18^, further indicating that the neck is not more sensitive than the palm in either ASD or TD. Thus, these findings taken together suggest that adults with ASD are not different from TD adults in the detection of light touch at various body sites. Anecdotally, individuals with ASD have reported that turtlenecks or clothing tags are uncomfortable. However, this does not seem to be caused by lower detection thresholds.

Third, results of the touch comparison task indicate that the ability to detect a slight difference between tactile stimuli in adults with ASD was also comparable to that of the controls. A previous study using simultaneous vibrotactile stimulations to two fingers has demonstrated that children with ASD showed higher discrimination thresholds than TD controls^21^. Puts, *et al*.^21^ argued that higher discrimination thresholds observed in ASD are due to the impairment of lateral inhibitory connections between minicolumns which reduce the contrast between brain areas representing different body sites. If that is true, in our task, successive stimulations to one body site that resulted in detection of a normal level in ASD might have been somewhat limiting, as testing of multiple body sites may have produced a different result. However, our task was different from Puts *et al*.’s task in many other aspects, such as the type of stimulus (static vs. dynamic), body sites (forearm vs. fingers), procedures (constant vs. up/down methods), and participants’ age (adults vs. children). Our results in the least indicate that the capacity for both detection and differentiation of small pressures on the skin are not substantially higher in ASD.

Tactile experiences involve both discriminative touch processing and affective responses. Most experimental studies have investigated hypersensitivity of ASD in relation to the former aspect in order to understand the mechanisms of tactile atypicality in ASD^16^, suggesting the involvement of peripheral mechanoreceptors such as Pacinian corpuscle^19^ and Meissner corpuscle^17^ and altered inhibitory functions in the central nerves^21–24^. However, these studies did not find relationships between laboratory findings and sensory difficulties in daily lives partly because the affective aspect has been relatively less studied. Thus, the current study provides new evidence of tactile atypicality in ASD by focusing on the affective aspect of touch and showing a relationship between increased autonomic response to tactile stimulation and sensory sensitivities in daily lives. Although the relationship between atypicalities of discriminative and autonomic processes should be further investigated, it is possible that atypical autonomic activity mediates the relationship between altered discriminative processing and tactile difficulties in daily lives. For instance, altered inhibitory function in central nerves causing poor detection of tactile stimuli and failure of adaptation might in parallel, or consequently, lead to excessive sympathetic nervous activity.

Tactile hypersensitivity in autonomic processes, but not subjective feelings, suggests that adults with ASD are not short on patience with tactile experiences. Rather, it is possible that their autonomic states respond excessively regardless of their conscious feelings. This view is supported by findings from a study using psychophysical tasks and functional magnetic resonance imaging^34^. Cascio, *et al*.^34^ demonstrated that subjective rating of adults with ASD to the roughness and pleasantness of some textured surfaces was not significantly different from that of controls, and that adults with ASD showed a greater neural response to an unpleasant tactile stimulus compared to the controls in affective somatosensory processing areas such as the posterior cingulate cortex and the insula. Their results therefore indicate that neural responses of adults with ASD are relatively hyperresponsive to unpleasant tactile stimulation. The current study agrees with this argument and provides complementary evidence in that the data show hypersensitivity to tactile stimuli in ASD in autonomic processing but not in subjective ratings of tactile stimuli. These data might reflect impaired interoceptive inference in ASD. In other words, individuals with ASD may fail to appropriately recognize their own emotional states, which might lead to excessive activation of the sympathetic nervous system as an indicator of exaggerated stress, anxiety, or panic^35^.

Moreover, there is some evidence of cellular and anatomical abnormalities that potentially explain atypicality in tactile sensation in ASD. C tactile (CT) afferents, which innervate hairy skin, are thought to be related to affective responses to tactile stimuli and pain inhibition^36–38^, and might be involved in activating some brain regions which have been implicated in ASD^39–41^. A small skin biopsy study investigating only four children with ASD reported that the number of small fibers, which presumably includes CT afferents, was fewer than usual; although it must be noted that the sample size was too small and there were no matched controls^42^. In addition, CT afferents responding to gentle touch, are activated by electrical stimulations^43^ but not vibrotactile stimulation^36,44–46^ and the activation of CT afferents evokes sympathetic skin response^46,47^. In the current study, although increased SCR to both strong and weak electrical stimulations was observed in ASD, only the latter was correlated with sensory difficulties in daily living. We thus speculate that regulation of the autonomic nervous system in response to tactile stimulation may be impaired in ASD due to dysfunction of processes involving CT afferents.

Some questions remain. First, we did not measure autonomic responses to the stimulation to hairless skin. Therefore, we cannot conclude that the excessive SCR reported herein is specific to hairy skin. A finding that excessive SCR in ASD is observed in hairy skin, but not hairless skin, would lend greater support to the involvement of dysfunctional CT afferents in ASD pathology. Second, the developmental factors and/or an innateness of tactile atypicality in ASD are still unknown. Studies investigating tactile responsiveness of school-aged children with ASD have provided mixed results from both behavioral and neural data^18,20,22,39,48,49^. A previous study measuring infants’ heart rate variability suggested that an atypical developmental trajectory in infancy predicted the severity of autistic traits^50^. Further study is needed to determine whether an atypical developmental trajectory of the autonomic nervous system would predict the extent of sensitivities to touch or other modalities.

Tactile experience is ubiquitous, and therefore modulates people’s daily lives, including social interactions such as intimate relationships or crowded situations. The current study demonstrates hypersensitivity to tactile stimuli in ASD in autonomic processes but not the perceptual processes. We propose the possibility that individuals with ASD build a unique way of social interaction based on their own sensory experiences, which might lead to impaired social interactions with TD individuals. Future study should investigate the developmental trajectory of social interaction in ASD with sensory atypicality taken into consideration.

## Methods

### Participants

Twenty-three adults with ASD participated in this study (12 males, 11 females; M age = 40.08 years, SD = 9.26, range = 25.42–59.75). Each of them was diagnosed by a clinical psychiatrist independently from our laboratory as having either autism spectrum disorder (n = 4), pervasive developmental disorder (PDD, n = 8), Asperger’s disorder/syndrome (n = 9), or PDD-not otherwise specified/-unspecified (n = 2) based on the DSM-5^14^, DSM-IV-TR^51^, or ICD-10^52^. Nineteen typically developing adults (TD) made up the study control group (9 males, 10 females; M age = 41.46 years, SD = 5.23, range = 33.25–49.58).

To examine autistic traits in both our ASD and control participants, we conducted the Japanese version of the Autism Diagnostic Observation Schedules (ADOS)^53^ on the participants with ASD. In addition, the Japanese version of the Social Responsiveness Scale, Second Edition (SRS-2)^54^, a self-report questionnaire used to identify the presence and severity of social impairment within the autism spectrum, was completed by all participants to provide further assessment of autistic traits. As expected, a subcategory of SRS-2, *Social Communication and Interaction*, was positively correlated with the corresponding subcategory in ADOS, sum of *Communication* and *Social Interaction* (*r* = 0.539, *p* = 0.014; Pearson’s correlation coefficient). The total SRS-2 score and scores of all subcategories were significantly higher in the ASD group than in the control group (total score, *t*(40) = 6.510, *p* < 0.001, see Supplementary Table S1 for further information). Note that only one male TD participant had a high total score (116) on the SRS-2 suggesting severe autistic symptoms. However, because the analyses after excluding him did not change any results in terms of statistical significance, the results reported herein included data for this participant.

Intelligence was assessed for all participants using the Japanese version of the Wechsler Adult Intelligence Scale, Third Edition (WAIS-III), except those who had had the test within five years in which case we asked the participants to show the test scores to us. Although FIQ of the ASD group was significantly higher than the TD group (*t*(40) = 2.748, *p* = 0.009), Cohen’s *d* = 0.85, and VIQ, *t*(40) = 3.839, *p* < 0.001, Cohen’s *d* = 1.19, PIQ was not different between the groups, *t*(40) = 0.791, *p* = 0.433, Cohen’s *d* = 0.25. In addition, the FIQ of all participants was above 70, indicating that no person with intellectual disabilities participated in the current study (for more detail, see Supplementary Table S1).

In order to estimate the quality of their experience with sensory stimuli in everyday life, all participants completed a self-report questionnaire, the Japanese version of the Adolescent/Adult Sensory Profile (AASP)^55^. Scores of three subcategories of four were significantly higher in the ASD group than the TD group: Poor Registration (*t*(40) = 5.913, *p* < 0.001), Sensitivity to Stimuli (*t*(40) = 4.000, *p* < 0.001), and Sensation Avoiding (*t*(40) = 2.715, *p* = 0.010). Another subcategory, Sensation Seeking, was significantly lower in the ASD group than in the TD group, *t*(40) = −4.308, *p* < 0.001. This pattern is in line with previous studies using the AASP^4,6^. Participant details are shown in Supplementary Table S1.

The participants with ASD were recruited from an autism research registry of the second author’s (S.K.) laboratory. The participants without ASD were recruited from a temporary staff service, stated that had not received diagnosis of potentially comorbid disorders, and gave consent to participate in the current study as typically developing individuals. All participants provided written informed consent, and were paid 1000 yen per hour for their time. All procedures were approved by the Research Ethics committee of the University of Tokyo (#15–74), and the study was conducted in accordance with standards specified in the 1964 Declaration of Helsinki.

### Design and target body sites

The protocol of the current experiment is outlined in Table 1. Each participant completed four tasks in the following fixed order: the touch comparison task (TCT), the touch detection task (TDT), the two-point discrimination task (2PDT), and the electrical stimulation task (EST). The entire experiment lasted approximately 2 hours. Participants were granted short breaks at any time (~15 min). In the TCT, the right dorsal forearm was the target site. In the TDT and the 2PDT, the right thenar palm, the right dorsal forearm, and the right half of the back of neck were the target sites. In the EST, the right ventral forearm was the target site. Because the TCT took the longest time, it was divided into four blocks. The TDT and the 2PDT were conducted between the blocks in the following order: the palm, the forearm, and the back of neck. The EST was conducted after finishing the three tasks.

**Table 1:** Protocol outline of the experiments.

TCT	TDT	2PDT	TCT	TDT	2PDT	TCT	TDT	2PDT	TCT	EST
1^st^ block	palm	palm	2^nd^ block	forearm	forearm	3^rd^ block	neck	neck	4^th^ block	forearm

Two questionnaires, SRS-2 and AASP, were completed before the tasks. Participants with ASD completed the ADOS and the WAIS-III on a different day. Participants without ASD were administered the WAIS-III on the same day that the tasks were conducted.

### Procedure

The experiment was conducted individually with each participant in a testing room. After the experimenter explained the current study and the participant provided informed consent, the tasks were carried out as described above. In all tasks, participants sat on a height-adjustable chair with their arms on a table in a comfortable position. Because we aimed to investigate participants’ tactile perception, we had to prevent the effects of visual cues. Therefore, during the three perceptual discriminative tasks (TCT, TDT, and 2PDT), a cardboard screen was put on the table between each participant’s arms and torso so that they could not see their hands and arms. The three perceptual discriminative tasks were programmed and run using MATLAB (version 7.12).

### Electrical Stimulation Task

#### Autonomic and subjective responses to electrical stimuli

To collect participants’ autonomic responses to electrical stimuli, each participants skin conductance response (SCR) was recorded throughout the whole task using a biomedical instrumentation system (EDA100C and MP150). A twin Ag/AgCl electrode (TSD203) with conductive gel (GEL101) was held on each participant’s right index and middle fingers using Velcro straps. Electrical stimuli were presented using a set of stimulator modules (STM100C and STMISOD), which was designed to limit the electrical power such that it did not convey any health risks. All recording of autonomic responses and electrical stimulations were controlled using dedicated software (AcqKnowlegde, all products described above, BIOPAC Systems, Inc., Goleta, CA).

For the electrical stimulations, a disposable twin Ag/AgCl electrode (EL-BAND, NIPRO, Japan) was attached to each participant’s right ventral forearm. The electrical stimulus was presented at 50 Hz lasting 1000 msec. We used the magnitude of electrical current as the intensity of stimuli and set it for each participant. Because the module could control the intensity of electrical stimulations with respect to voltage but not current, we determined voltages that corresponded to electrical currents of 200–500 μA as a weak stimulus and 1000–2000 μA as a strong stimulus for each participant before starting the task. To achieve this, and to avoid a sudden intense stimulation, each participant was presented with 1000-msec stimuli of gradually increasing intensity from subthreshold levels in steps of 0.25 volts. If a participant refused further stimulation before determining the weak stimulus, the task was terminated (one male participant with ASD gave up the task for this reason). In addition, if a participant reported that their tolerance was exceeded before the stimulation reached 1000 μA, the intensity at that point was set as the strong stimulus.

After determining the stimulus intensity for weak and strong conditions for each participant, the main task started. In the main task, the weak and strong stimuli were repeated alternately three times in a fixed order (i.e., weak, strong, weak, strong, weak, and strong). Participants were asked to relax throughout the main task. Each electrical stimulus was preceded by 3000-msec resting period and then presented for 1000 msec. Immediately after each stimulation, participants were asked to orally report their subjective responses when an experimenter presented a sheet with scales from 0 (not at all) to 10 (extremely) (i.e., 11 grades) for “Intensity,” “Painful,” and “Unpleasant.” After a resting period of approximately 40 sec, the next stimulus was presented. During trials, we monitored and recorded the magnitude of electrical current presented to participants as well as SCR so that we could analyze the data offline.

#### Sensation and pain thresholds

To assess participants’ sensation and pain thresholds to electrical stimulation, we used an automated pain evaluation system used for medical purposes (PainVision, OSACHI Co., Ltd., Japan). A disposable twin Ag/AgCl electrode (EL-BAND, NIPRO, Japan) was attached to each participant’s right ventral forearm. Before beginning the assessments, participants experienced electrical stimulation of 50 Hz from this device and were instructed on how to use the device. Then, to assess the perception threshold, we measured the minimum current that participants could perceive by gradually increasing the stimulating current. Participants were told to push a button at the moment when they felt any electrical stimulation around their right ventral forearm. When they pushed the button, the stimulation stopped. The average current across three trials was defined as the perception thresholds. After that, we measured the minimum current that elicited pain. The electrical stimulation was gradually increased in the same way as the perception threshold, and participants were told to push the button at the moment they felt any pain around their ventral right forearm. The average of current across three pain stimulation trials was defined as the pain threshold for each participant.

### Two-Point Discrimination Task

We made a set of twin thin needles with round tips to investigate the threshold force for two-point touch detection. The ranges of the two points were varied from 2 to 90 mm (at 1 mm intervals from 2 to 20 mm, 2 mm intervals from 20 to 40 mm, and 5 mm intervals from 40 to 90 mm), and also used a single needle (i.e., zero-mm range). Adopting the up-down method, two consecutive correct trials were followed by a trial with a decreased range (more difficult) and one incorrect trial was followed by a trial with an increased range (easier). The task ended if reversals of the ranges of presentation occurred ten times or if ten consecutive correct trials occurred at the smallest range (2 mm) (Five participants passed the smallest range for the palm: 3 in the ASD group and 2 in the TD group). Each trial consisted of successive two-point and one-point presentations. The order of the two- and one-point presentations was counterbalanced across trials. In each trial, verbal prompts were given “A” before the first presentation, and then “B” before the second presentation. Participants were told to report which presentation was a two-point stimulus by saying “A” or “B” after the second presentation. Thresholds were calculated by averaging the values of the last five reversal trials.

### Touch Detection Task

We investigated the threshold force for touch detection using a set of “von Frey” monofilaments, of which the forces were 0.008, 0.02, 0.04, 0.07, 0.16, 0.4, 0.6, 1.0, 1.4, 2.0, 4.0, and 6.0 grams (EXACTA Touch Test Sensory Evaluators, North Coast Medical, Inc., Gilroy, CA). Adopting the up-down method, two consecutive correct trials were followed by a trial with a decreased force (more difficult) and one incorrect trial was followed by a trial with an increased force (easier). The task ended if reversals of the forces of presentation occurred ten times or if ten consecutive correct trials occurred in the smallest force (0.008 gram). Each trial consisted of successive stimulated and non-stimulated (i.e., a catch) presentations. The order of the stimulated and non-stimulated presentations was counterbalanced across trials. In each trial, verbal prompts were given “A” before the first presentation, and then “B” before the second presentation. Participants were told to report which presentation was actually stimulated by saying “A” or “B” after the second presentation. Thresholds were calculated by averaging the values of the last five reversal trials.

### Touch Comparison Task

To investigate participants’ sensitivity to the difference of touch forces, we used a set of seven calibrated nylon “von Frey” monofilaments, of which the forces were 0.4, 0.6, 1.0, 1.4, 2.0, 4.0, and 6.0 grams. Adopting the constant method with two interval first choice task to minimize response bias, the middle force monofilament (1.4 grams) was presented as the standard stimulus and the others as the comparison stimuli. Because this method required many trials, the trials were divided into four blocks. Each block included eight trials for each comparison stimulus and 48 trials in total. Thus, each participant experienced 32 trials for each comparison stimulus and 192 trials in total across the four blocks. Each trial consisted of successive presentations of the standard stimulus and one of the comparison stimuli. The order of the presentations of the standard and the comparison stimuli within a trial was counterbalanced across trials. For each stimulus, the experimenter manually lowered the monofilament to the right dorsal forearm until the tip of made a contact with the skin and a continuous force was applied smoothly until the monofilament buckled once. The contact area was marked by an adhesive plaster with its center cut out one centimeter square. In each trial, the first and second presentations followed verbal prompts “A” or “B” respectively. Participants were told to report which stimulus was strong by saying “A” or “B” after the second presentation.

## Electronic supplementary material

Supplementary information
