# Publisher Correction: Autonomic versus perceptual accounts for tactile hypersensitivity in autism spectrum disorder

**DOI:** 10.1038/s41598-017-17125-3

**Published:** 2017-12-05

**Authors:** Hiroshi Fukuyama, Shin-ichiro Kumagaya, Kosuke Asada, Satsuki Ayaya, Masaharu Kato

**Affiliations:** 1 Center for Baby Science, Doshisha University, Kizugawa, Japan; 2 Graduate School of Arts and Sciences, The University of Tokyo, Tokyo, Japan; 3 Research Center for Advanced Science and Technology, The University of Tokyo, Tokyo, Japan

*Scientific Reports* **7**:8259; doi:10.1038/s41598-017-08730-3; Article published online 15 August 2017

In the original version of this Article, the affiliation for Shin-ichiro Kumagaya was omitted. The correct affiliation for Shin-ichiro Kumagaya is given below:

Research Center for Advanced Science and Technology, The University of Tokyo, Tokyo, Japan.

In addition, this Article contained errors in Reference 9, which was incorrectly given as:

“Marco, E. J., Hinkley, L. B., Hill, S. S. & Nagarajan, S. S. (Nature Publishing Group, 2011)”.

These errors have now been corrected in the PDF and HTML versions of the Article.

