# Supplementary material for: Autonomic versus perceptual accounts for tactile hypersensitivity in autism spectrum disorder
Source: Sci Rep. 2017 Aug 15;7:8259. doi: 10.1038/s41598-017-08730-3 (PMC5557757; doi:10.1038/s41598-017-08730-3)
Supplement: Supplementary file 1 — Supplementary information [file 41598_2017_8730_MOESM1_ESM.pdf]

Autonomic versus perceptual accounts for tactile hypersensitivity in autism spectrum disorder

Hiroshi Fukuyama[1,2]

Shin-ichiro Kumagaya[3]

Kosuke Asada[3]

Satsuki Ayaya[3]

Masaharu Kato[1]

[1] Center for Baby Science, Doshisha University, Japan

[2] Graduate School of Arts and Sciences, The University of Tokyo, Japan

[3] Research Center for Advanced Science and Technology, The University of Tokyo, Japan

Supplementary information

Figure S1. The mean value of the slope across groups in the touch comparison task

Figure S2. The mean fraction of strong response to each comparison stimulus compared to the standard stimulus across groups in the touch comparison task

Table S1. Profile data of the participants in each group

Supplementary information

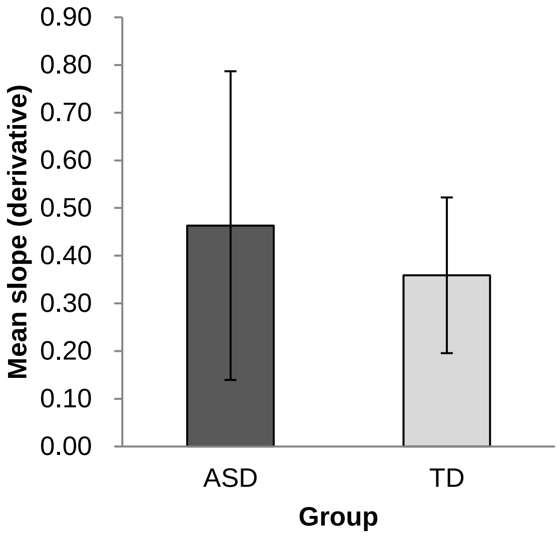

Figure S1. The mean value of the slope (derivative) across groups. A steeper slope means more sensitivity to force differences in light touch (monofilament stimuli). Error bars show SD.

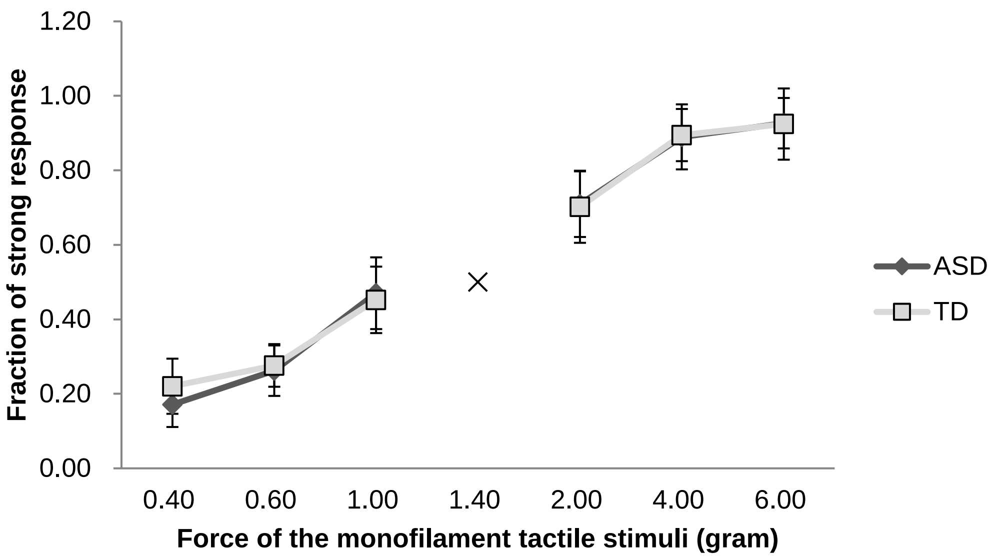

Figure S2. The mean fraction of strong response to each comparison stimulus compared to the standard stimulus (1.40 g) across groups. X shows the force of the standard stimulus and was not included in the comparison stimuli. Error bars show SD.

Table S1. Profile data of the participants in each group

			ASD (*n* = 23, 12 males)		TD (*n* = 19, 9 males)	
			Mean (SD)	range	Mean (SD)	range
Age (year)			40.08 (9.26)	25.42-59.75	41.46 (5.23)	33.25-49.58
WAIS-III	FIQ		111.957 (15.665)	78-136	99.947 (11.895)	71-116
	VIQ		120.217 (14.155)	96-144	103.316 (14.256)	70-127
	PIQ		99.174 (18.067)	62-135	95.579 (8.840)	78-109
ADOS (*n* = 20)			8.35 (3.99)	1-16	-	-
SRS-2	Total		108.83 (29.22)	50-157	56.58 (21.11)	24-116
	SCI		87.30 (23.93)	40-128	47.79 (16.52)	20-93
		Awr	11.04 (3.01)	4-16	6.89 (2.42)	3-13
		Cog	21.17 (5.19)	11-30	10.79 (3.71)	6-21
		Com	36.43 (11.61)	13-54	17.84 (8.57)	7-41
		Mot	18.65 (6.59)	10-30	12.26 (4.42)	4-20
	RRB		21.52 (6.37)	10-31	8.79 (5.63)	2-23
AASP	Poor Registration		43.04 (7.74)	27-53	28.74 (7.89)	19-45
	Sensation Seeking		32.65 (7.59)	18-46	41.74 (5.69)	33-53
	Sensitivity to Stimuli		47.61 (11.42)	28-68	35.26 (7.79)	22-54
	Sensation Avoiding		44.83 (11.21)	26-68	36.26 (8.74)	25-54

ASD, autism spectrum disorder; TD, typically developing; WAIS-III, Wechsler Adult Intelligence Scale, third edition; FIQ, Full Intelligence Quotient; VIQ, Verbal IQ; PIQ, Performance IQ; ADOS, Autism Diagnostic Observation Schedule (The ADOS was not conducted for the TD group.); SRS-2, Social Responsiveness Scale, second edition (Japanese version); SCI, Social Communication and Interaction; Awr, Social Awareness; Cog, Social Cognition; Com, Social Communication; Mot, Social Motivation; RRB, Restricted Interests and Repetitive Behavior; AASP, Adolescent/Adult Sensory Profile (Japanese version)
